# Robotic-assisted radical cystoprostatectomy and intracorporeal ileal conduit formation in a patient with prior total colectomy and J-pouch

**DOI:** 10.1093/jscr/rjaf1104

**Published:** 2026-01-21

**Authors:** Grace S Sutherland, Jodie M McDonald, Amanda C Chung, Justin V Vass, Ahmed G Goolam, Matthew R Winter

**Affiliations:** Department of Urology, Royal North Shore Hospital, Sydney, NSW 2065, Australia; Department of Urology, Royal North Shore Hospital, Sydney, NSW 2065, Australia; Department of Urology, Royal North Shore Hospital, Sydney, NSW 2065, Australia; Department of Urology, Royal North Shore Hospital, Sydney, NSW 2065, Australia; Department of Urology, Royal North Shore Hospital, Sydney, NSW 2065, Australia; Department of Urology, Royal North Shore Hospital, Sydney, NSW 2065, Australia

**Keywords:** bladder cancer, ileal conduit, J-pouch, robotic surgery, radical cystectomy

## Abstract

We present the first reported case of robotic-assisted radical cystoprostatectomy and ileal conduit formation in a patient with prior total colectomy and J-pouch. The anatomy posed significant technical risk due to end artery pouch perfusion and changes in the posterior plane. Intraoperative strategies, including rectal indocyanine green and a short discard segment when constructing the conduit enabled successful outcomes. The procedure was completed without complication, and the patient had a favourable oncologic and functional status postoperatively. This case demonstrates the technical considerations of cystectomy in patients with previous large bowel resection and reconstruction, and the outlined strategies are relevant across multiple clinical settings.

## Introduction

Previous surgery alters vascular anatomy creating challenging conditions in subsequent pelvic surgery. In patients who have undergone large bowel surgery necessitating ligation of the inferior mesenteric artery, collateral blood supply is disrupted [1]. Following total proctocolectomy with ileal pouch–anal anastomosis (J-pouch), perfusion of the J-pouch depends primarily on the superior mesenteric artery, particularly the ileocolic branch [2]. The absence of collateral supply leaves the J-pouch reliant on end arteries, placing it at high risk of revascularization [2]. These anatomical changes, combined with dense adhesions and scarring from prior surgery can create a challenging environment during any subsequent pelvic surgery [3].

Radical cystectomy with pelvic lymph node dissection for muscle-invasive bladder cancer remains the standard of care for achieving long-term disease control in organ-confined disease [4]. Urinary diversion is achieved commonly via ileal conduit formation [5]. Robotic-assisted techniques are now widely adopted in urological surgery, providing enhanced vision, precision, and ergonomics [6].

To our knowledge, this is the first reported case of robotic radical cystectomy in a patient with prior total colectomy and J-pouch. These patients present a uniquely high-risk scenario due to end-artery pouch perfusion without arterial collateralization and altered pelvic anatomy. Together, these factors significantly increase the risk of unintentional pouch revascularization, making successful robotic dissection and preservation of pouch integrity technically demanding.

## Case presentation

**Figure 1:**
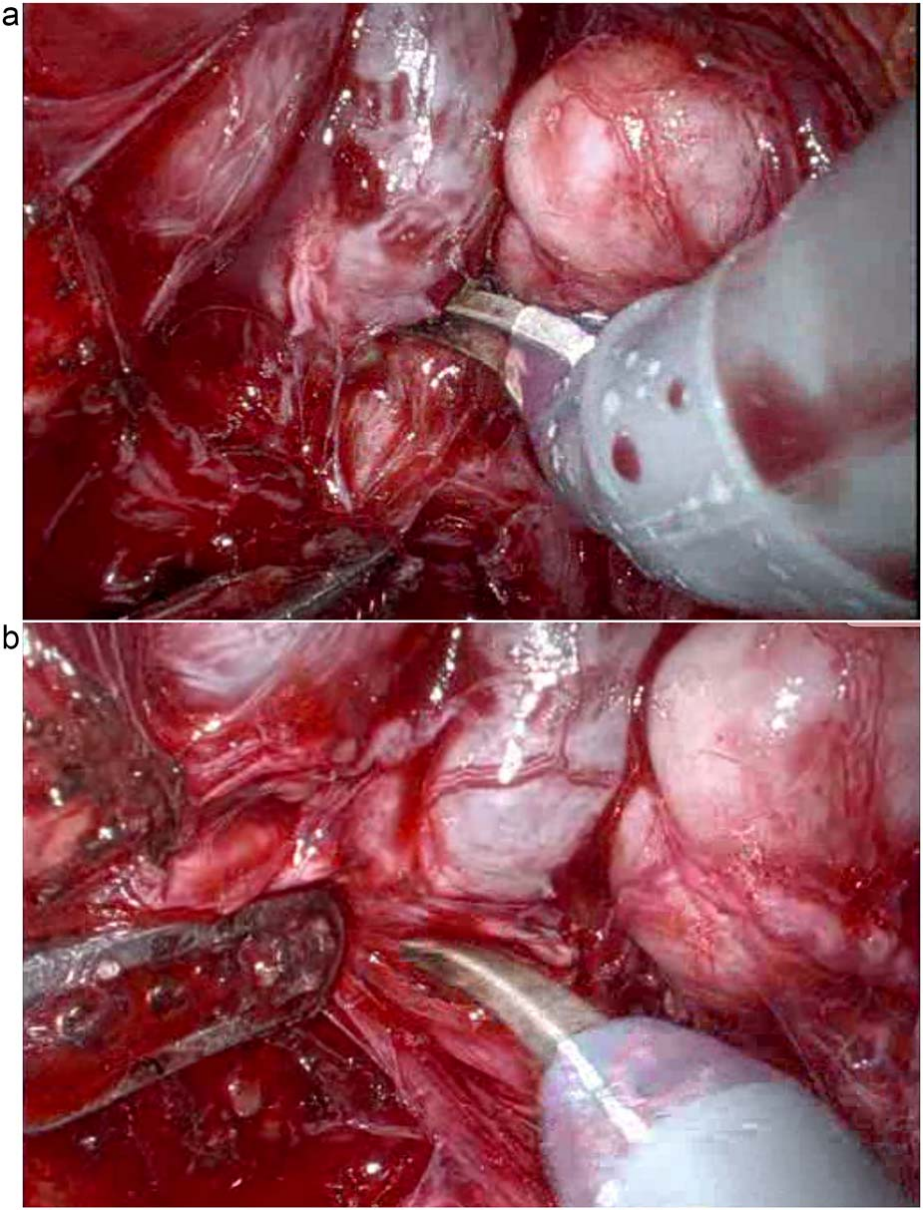
Posterior pelvic dissection demonstrating the challenging plane in the absence of normal perirectal fat (a and b).

**Figure 2:**
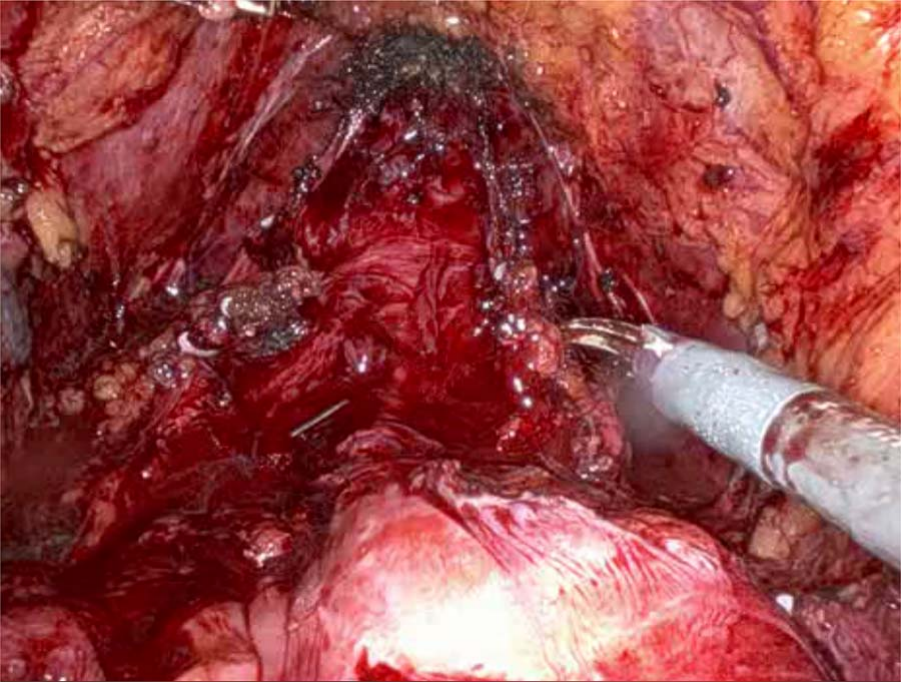
Intraoperative view showing the J-pouch after removal of the bladder and prostate.

A 58-year-old man with a history of familial adenomatous polyposis and previous total colectomy and J-pouch presented with muscle-invasive bladder cancer. Following four cycles of neoadjuvant chemotherapy, he underwent robotic-assisted radical cystoprostatectomy, bilateral pelvic lymph node dissection, and ileal conduit formation. Surgical entry was achieved via a modified Hasson technique and robotic port placement. Careful dissection, particularly in the posterior plane (Fig. 1), was required due to altered pelvic anatomy. Indocyanine green was injected directly into the J-pouch via a rectal tube to access perfusion and anatomical planes. Subsequent ligation with vessel sealer of the vascular pedicles was performed, and the bladder and prostate were removed en bloc (Fig. 2). Extensive adhesiolysis was necessary to mobilize sufficient small bowel to form the conduit and reach the anterior abdominal wall without tension. A 15 cm segment of small bowel proximal to the J-pouch was isolated using an Echelon stapler, and a distal 5-cm discard segment was taken to ensure adequate distal perfusion of the conduit and aid mobility for stoma formation. Bowel continuity was restored with a stapled side to side anastomosis. The ileal conduit was formed intracorporeally and delivered through the abdominal wall.

Indocyanine green was given intravascularly to assess ureteric perfusion, with excision of any distal devascularized segments. The ureters were then anastomosed to the conduit using the Bricker technique with interrupted 4–0 vicryl sutures. Haemostasis was achieved with vessel sealer, with minimal clipping and suturing near the J-pouch. There were no intraoperative complications, and total estimated blood loss was 150 ml.

Postoperative recovery was uncomplicated. Bowel function returned on postoperative day four. Ureteric stents and surgical drains were removed on Day 6, and the patient was discharged home on Day 7. Final histopathology showed complete treatment response (pT0) of urothelial carcinoma with clear surgical margins and incidental ISUP grade group 3 prostate adenocarcinoma.

## Discussion

Robotic-assisted radical cystectomy with intracorporeal ileal conduit formation in patients with prior total colectomy and J-pouch reconstruction involves significant technical difficulty and vascular risk due to dense adhesions and the pouch’s reliance on end-artery perfusion. The changes in the posterior plane secondary to the loss of the usual perirectal fat and denonvilliers fascia, as well as the pouch being comprised of thin walled small bowel, make this dissection particularly challenging (Fig. 1). Additionally, remaining length of small bowel has a functional consideration and short gut syndrome has to be considered. To our knowledge, this is the first reported case of such a procedure.

Few reports describe pelvic robotic surgery following major colorectal procedures. Siddiqui *et al*. found that among patients undergoing robotic prostatectomy, those with prior colectomy had the highest rates of adhesiolysis and the longest operative times compared to all other types of previous abdominal surgery [7]. Mustafa *et al*. concluded that robotic radical prostatectomy is feasible and safe after previous colorectal surgery, despite increased technical complexity due to altered pelvic anatomy [8]. Watanabe *et al*. described a case of robotic cystectomy following low anterior resection, in which dense adhesions from prior surgery significantly complicated pelvic dissection [3].

Other studies support the feasibility of robotic pelvic surgery in patients with prior abdominal operations. Yuh *et al*. found that while previous lower abdominal surgery increased postoperative complications, it did not prevent successful robotic completion [9]. Similarly, Park *et al*. showed that prior abdominal surgery, including gynaecologic procedures, did not adversely affect outcomes in robotic colorectal surgery [10].

## Conclusion

This case demonstrates that robotic cystoprostatectomy with intracorporeal ileal conduit formation is technically achievable in patients with prior total colectomy and J-pouch. With appropriate intraoperative strategies, such as direct indocyanine green injection into the J-pouch and a distal discard segment when constructing the conduit, safe dissection, and pouch preservation can be accomplished despite complex pelvic anatomy and vascular constraints.

### Learning points

- Prior colectomy and J-pouch reconstruction significantly alter pelvic vascular anatomy.
- Direct indocyanine green injection into the pouch helps delineate planes and assess perfusion.
- A distal discard segment during conduit isolation aids conduit mobility and perfusion.
- Robotic techniques enable precise dissection in anatomically distorted pelvises.

## References

[1] Sakorafas GH, Zouros E, Peros G. Applied vascular anatomy of the colon and rectum: clinical implications for the surgical oncologist. Surg Oncol 2006;15:243–55. 17531744 10.1016/j.suronc.2007.03.002

[2] Helavirta J, Haapamäki MM, Koskensalo S, et al. Vascular considerations in ileal pouch surgery: a review. Tech Coloproctol 2020;24:935–42.32385673

[3] Watanabe S, Kobayashi H, Hiroe N, et al. Robot-assisted radical cystectomy for bladder cancer after low anterior resection: a case report. Asian J Endosc Surg 2024;17:e13345. 38943367 10.1111/ases.13345

[4] Patel VG, Oh WK, Galsky MD. Treatment of muscle-invasive and advanced bladder cancer in 2020. CA Cancer J Clin 2020;70:404–23. 32767764 10.3322/caac.21631

[5] Lee RK, Abol-Enein H, Artibani W, et al. Urinary diversion after radical cystectomy for bladder cancer: options, patient selection, and outcomes. BJU Int 2014;113:11–23. 24330062 10.1111/bju.12121

[6] Rassweiler JJ, Autorino R, Klein J, et al. Future of robotic surgery in urology. BJU Int 2017;120:822–41. 28319324 10.1111/bju.13851

[7] Siddiqui SA, Krane LS, Bhandari A, et al. The impact of previous inguinal or abdominal surgery on outcomes after robotic radical prostatectomy. *Urology* 2010;75:1079–82. 19896178 10.1016/j.urology.2009.09.004

[8] Mustafa M, Pettaway CA, Davis JW, et al. Robotic or open radical prostatectomy after previous open surgery in the pelvic region. Korean J Urol 2015;56:131–7. 25685300 10.4111/kju.2015.56.2.131 PMC4325117

[9] Yuh BE, Ciccone J, Chandrasekhar R, et al. Impact of previous abdominal surgery on robot-assisted radical cystectomy. *JSLS* 2009;13:398–403. 19793483 PMC3015968

[10] Park S, Kang J, Park EJ, et al. Laparoscopic and robotic surgeries for patients with colorectal cancer who have had a previous abdominal surgery. Ann Coloproctol 2017;33:184–9. 29159166 10.3393/ac.2017.33.5.184 PMC5683969

